# Hemichorea as a primary manifestation of varicella-zoster virus neuroinfection in a child - a case report and review of literature

**DOI:** 10.1007/s10072-025-08550-8

**Published:** 2025-10-11

**Authors:** Mateusz Tomkiewicz, Dominik Gosławski, Zuzanna Krasula, Agata Klawikowska, Marta Zawadzka, Jakub Szymarek, Maria Mazurkiewicz-Bełdzińska

**Affiliations:** Department of Developmental Neurology, Medical University of Gdansk, Gdansk, 80-210 Poland

**Keywords:** Chickenpox, Varicella-zoster, Chorea, Hemichorea, Extrapyramidal signs, Stroke in children, Vasculitis

## Abstract

**Background:**

Varicella is a common childhood infection caused by the varicella-zoster virus. Reactivation of latent virus within somatosensory ganglia can lead to numerous complications, including cerebral vasculitis. Inflammatory processes can alter vascular structure and lead to ischemic stroke in both the pediatric and adult population. Chorea is a rare extrapyramidal symptom which might be a result of vascular insult in the course of cerebrovascular events.

**Case presentation:**

An 8-year old boy with a recent history of varicella presented with acute-onset choreiform movements of the right extremities. A lumbar puncture revealed the presence of varicella-zoster virus genetic material in the cerebrospinal fluid. Brain magnetic resonance imaging showed ischemic lesions affecting basal ganglia, while vascular imaging was unremarkable. A diagnosis of arterial ischemic stroke secondary to varicella-zoster vasculitis was suspected, which was later supported by exclusion of other potential causes of cerebrovascular events. After a course of acyclovir and prednisone, as well as the initiation of a long-term antiplatelet therapy, marked clinical recovery was achieved. A follow-up visit 6 months later confirmed our patient’s return to his pre-stroke level of functioning.

**Conclusions:**

Varicella remains a major cerebrovascular risk factor, particularly in the unvaccinated pediatric population. Patients with varicella-zoster vasculitis might present with various neurological deficits, including hemichorea, even when vascular imaging reveals no abnormalities. Early initiation of antiviral, steroid and antiplatelet treatment might positively impact the clinical recovery time of affected individuals, although a need for large-cohort studies assessing treatment efficacy is evident.

## Background

Varicella, also known as chickenpox, is a common childhood infection caused by varicella-zoster virus (VZV), a highly contagious member of the Herpesviridae family, transmitted mainly by airborne droplets. The symptoms of the disease usually appear 10 to 21 days after exposure and include a generalized, rapidly evolving, pruritic skin rash forming small blisters and pustules. Skin lesions are often accompanied or preceded by fever, fatigue, pharyngitis and headaches [1–3]. 

Chickenpox can lead to many complications, including bacterial superinfection of skin lesions, thrombocytopenia, glomerulonephritis, arthritis, hepatitis and primary viral pneumonia [4]. Some of them are caused by reactivation of VZV from sensory ganglia. The most common manifestation of reactivation is herpes zoster (HZ), which is often complicated by postherpetic neuralgia. Other neurological complications include meningoencephalitis, meningoradiculitis, myelopathy and cerebellitis [5]. VZV is also a rare causative agent of cerebral vasculopathy, which may present itself as vascular structural alterations (dissection, aneurysm formation, moyamoya-like syndrome), as well as vasculitis [6–9]. VZV vasculitis is an inflammatory process within the vessel wall which can lead to vascular remodeling and cerebrovascular events, such as ischemic or hemorrhagic stroke [4, 10]. Vasculopathy in the course of VZV infection may have various presentations, such as hemiparesis, vision loss, imbalance, as well as other focal neurological deficits [8, 11, 12]. 

Chorea is a hyperkinetic movement disorder characterized by the presence of involuntary, flowing, unpredictable movements. Its etiology is varied and includes autoimmune, infectious, vascular, metabolic, genetic or iatrogenic causes. It may occur both in adults and children [13]. 

Hereby, we present a case report of an 8-year-old patient who developed hemichorea as the main clinical manifestation of VZV cerebral vasculitis.

## Case presentation

An 8-year-old boy, with no significant prenatal or perinatal history and normal developmental milestones, was admitted to the Developmental Neurology Clinic from a Paediatric Ward due to choreiform dyskinesia of the right extremities.

The patient had an episode of uncomplicated varicella in December 2023, with no additional symptoms reported. He has not been vaccinated against it, however. Moreover, he experienced an episode of febrile seizures around the age of 3.

In June 2024, the patient’s parents noticed a presence of involuntary movements of twisting quality, mainly in the right upper limb. Motor agitation had been observed since the onset of symptoms. Moreover, for the past month the patient had complained of headaches on three occasions, with vomiting accompanying the headache episodes twice. Over the past two months, the patient had received no vaccinations and had shown no evidence of infection, exposure to chemicals, medications, or psychoactive substances.

The patient was first admitted to a Paediatric Ward on the 10th of June. During the inpatient stay, several diagnostic tests were performed; the Computed Tomography (CT) scan revealed no acute abnormalities, serologic testing for Lyme disease was negative, cerebrospinal fluid (CSF) analysis showed pleocytosis of 35 cells/µL, with no deviations in protein concentrations. Polymerase Chain Reaction (PCR) analysis of the CSF detected VZV genetic material; consequently, the patient was started on intravenous acyclovir. After three doses of the medication, a reduction in the severity of involuntary movements was observed.

At presentation at the Developmental Neurology Clinic on the 12th of June, the patient was in a good general condition, was able to communicate clearly and logically, and circulatory and respiratory functions were unremarkable. He exhibited fluctuating involuntary movements affecting the right upper limb and, episodically, the right lower limb, particularly during ambulation. The movements were mainly of choreiform character, with intermixed athetotic qualities. They were arrhythmic and variable in intensity, exacerbated in an elevated position. No Babinski sign was elicited bilaterally, no meningeal signs were present, and the Romberg test was negative. Additionally, transient drooping of the right corner of the mouth due to a right-sided central facial nerve palsy, tongue deviation to the right, and slurred speech were observed.

**Fig. 1:**
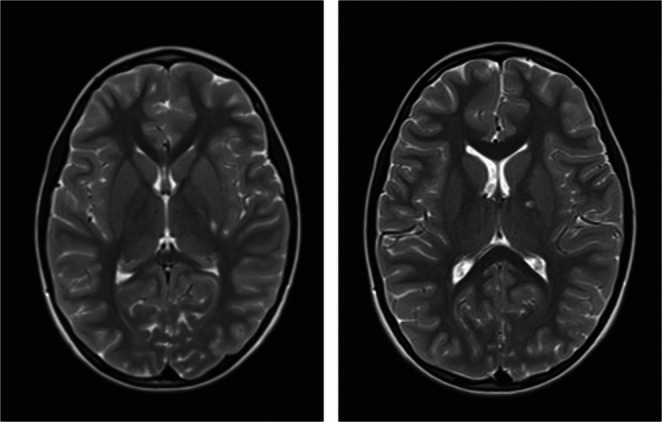
Brain MRI image. Diffuse vascular changes in the left hemisphere, namely in the left putamen and posterior limb of the left internal capsule

Additionally, Magnetic Resonance Imaging (MRI) with vascular imaging was conducted (Fig. 1). It revealed diffuse vascular changes in the left hemisphere of the brain, subacute and chronic in nature. Several small (up to 5 mm) hyperintense foci on T2-weighted images/FLAIR were detected within the white matter of the left hemisphere. They were also present in the deep cerebral structures, namely the internal capsule and putamen. No other encephalic abnormalities were identified and the vascular imaging was unremarkable. A diagnostic workup for stroke and vasculitis was implemented as the latter was suspected.

As part of the diagnostic workup, metabolic, nutritional, and thyroid abnormalities were ruled out. There was no evidence of systemic autoimmune diseases or prothrombotic state, including thrombophilia. In the following days, serological testing for varicella from blood samples indicated positive Immunoglobulin G (IgG) and marginally negative Immunoglobulin M (IgM). Moreover, after a cardiological consultation and diagnostic workup, cardiogenic causes were excluded.

Besides acyclovir, the patient was also prescribed oral prednisone (1 mg/kg body weight. for 5 days) and acetylsalicylic acid (ASA) 1x a day 75 mg, as a prophylactic anticoagulation dose.

During the hospitalization, the symptoms showed gradual improvement with treatment and by the end of the stay, the involuntary movements were minimal. 12 days after admission, the patient was released in good condition with a recommendation to maintain ongoing ASA treatment until the next visit, whereas the acyclovir treatment was stopped.

On a follow-up visit 3 months later, no focal neurological signs were observed. The patient was scheduled for a cranial MRI which demonstrated partial regression of previously described foci. According to the parents and the patient, the involuntary movements and headaches have subsided since the last inpatient stay.

**Fig. 2:**
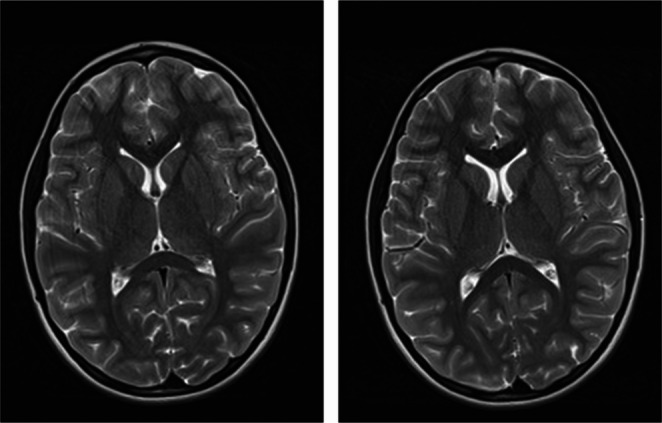
Brain MRI image. Regression of observed lesions. Solitary small focus found in the posterior region of the left putamen

**Fig. 3:**
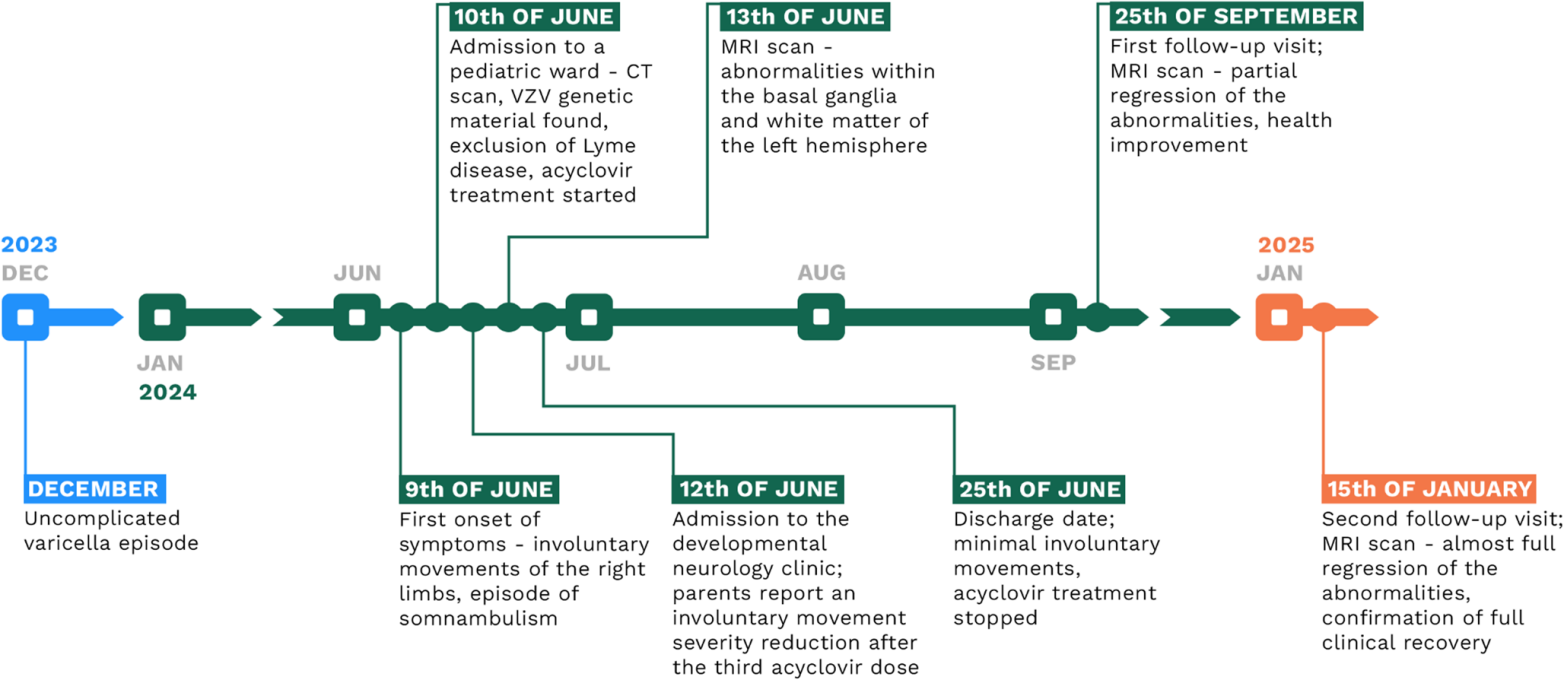
Timeline summary

A visit on the 15th of January 2025 confirmed subsequent regression of the vascular abnormalities with only a solitary small focus found in the posterior region of the left putamen (Fig. 2). According to the patient and his parents, all previously reported symptoms have completely resolved. He has returned to his baseline level of health and neurological function, indicating a full recovery. The cessation of ASA treatment was recommended (Fig. 3).

The patient and his parents provided consent to share their experience with the authors, contributing valuable perspective on the illness course and effect of the treatment. They also authorized the publication of diagnostic and therapeutic measures described above.

### Literature review

We have conducted a review of studies regarding VZV-vasculitis. A search of Embase, Medline and Scopus databases with combinations of terms “VZV”, “varicella-zoster”, “varicella”, “chickenpox”, combined with “vasculitis”, “vasculopathy”, “angiopathy”, “stroke”, was made. Among searched results, only 23 contained reports of acute-onset chorea as a consequence of vasculitis following primary VZV infection (Table 1). The median age at symptom onset was 4, which is consistent with reported VZV seropositivity among different age groups [14]. Out of 5 patients for whom vaccination status was reported, none were vaccinated against VZV.

**Table 1:** Comparison of published cases of VZV-vasculitis-related chorea and our patient showing diverse presentations, different diagnostic measures, methods of treatment and its effects. The table has been arranged chronologically, in order of publication date

no.	Authors	Age, sex	Affected side	Time from VZV infection to hemichorea	Varicella vaccination status	Other clinical manifestations	Lesion location (MRI)	Affected vessels (MRA)	Side of described lesions	Laboratory findings - serum	Laboratory findings - CSF	Treatment	Clinical recovery time
1	Klaus P Hammann et al. [15]	20, F	R	2 weeks	not available (N/A)	myoclonic-like movements of arm and face paraesthesiae of fingers motor aphasia pyramidal syndrome	N/A	N/A	N/A	(+) anti-VZV IgG	low protein content	carbamazepine	N/A
2	Silerstein et al.[16]	6	R	2–3 months	N/A	facial weakness hemiparesis	globus pallidus caudate nucleus frontal lobe	ICA ACA (A1) MCA (M1)	L	(-) anti-VZV IgG	9 white blood cells/mm^3^	glucocorticoids	N/A
3	Miravet et al.[17]	4	N/A	N/A	N/A	transient hemifacial weakness	basal ganglia white matter	MCA	N/A	N/A	N/A	N/A	N/A
4	Miravet et al.[17]	5	L	N/A	N/A	hemiparesis	basal ganglia	MCA ACA	N/A	N/A	N/A	N/A	N/A
5	Buompadre et al.[18]	4, F	N/A	N/A	N/A	hemiparesis	lentiform nucleus caudate nucleus internal capsule	MCA (M1)	N/A	N/A	N/A	N/A	N/A
6	Bartolini et al.[19]	3, M	R	15 days	N/A	none	caudate nucleus lenticular nucleus	MCA	L	(+) IgG and IgM Ab against VZV (+) anti-protein S IgG and IgM antibodies	N/A	ASA	N/A
7	Bartolini et al.[19]	4, F	R	7 months	N/A	none	caudate nucleus lenticular nucleus internal capsule	ACA (A1) MCA (M1)	L + R	(+) IgG Ab against VZV (-) IgM Ab against VZV	N/A	ASA valproic acid	N/A
8	Bauder et al.[20]	6,F	N/A	N/A	N/A	headache	N/A	MCA PCA	N/A	N/A	(+) VZV-DNA PCR	acyclovir glucocorticoids	N/A
9	Hackenberg et al.[21]	4, M	N/A	N/A	N/A	gait disorder dysphasia facial weakness mood disorder	putamen	ICA MCA ACA	R	N/A	(-) VZV-DNA PCR	acyclovir ASA glucocorticoids cyclophosphamide	N/A
10	Bulder et al. [22]	2, M	R	2 months	N/A	none	caudate nucleus putamen	ICA ACA MCA (M1-M3)	L	N/A	(-) VZV-DNA PCR	ASA	1 month
11	Bulder et al. [22]	3, F	L	7 months	N/A	none	caudate nucleus putamen	MCA ACA	R	N/A	N/A	ASA	3 weeks
12	Spagnoli et al. [23]	5, F	L	5 months	unvaccinated	trigeminal autonomic pain vomiting hemifacial grimaces lingual dyskinesias hypotonia hyposthenia exaggerated knee reflex	lentiform nucleus internal capsule caudate nucleus	ACA (A1)	R	(+) IgG Ab against VZV	N/A	acyclovir ASA valproic acid	N/A
13	Davico et al.[24]	3, M	R	6 months	N/A	none	lentiform nucleus internal capsule	none	L	(+) anti-VZV IgG	N/A	ASA prednisone haloperidol	3 weeks
14	Davico et al.[24]	3, M	L	4 months	N/A	none	thalamus internal capsule	ICA MCA	R	(+) anti-VZV IgG	N/A	ASA prednisone haloperidol	3 weeks
15	Helmuth et al.[25]	4, M	R	8 months	N/A	hemiparesis	basal ganglia	none	L	N/A	(+) VZV-DNA PCR (+) IgG Ab against VZV	acyclovir ASA glucocorticoids	N/A
16	Helmuth et al.[25]	5, M	R	6 months	N/A	dysarthria	temporal lobe	ICA	L	N/A	(+) VZV-DNA PCR (+) IgG Ab against VZV	acyclovir ASA glucocorticoids	N/A
17	Helmuth et al.[25]	5, M	L	4 months	N/A	hemiparesis	white matter	MCA ACA ICA	R	N/A	(+) VZV-DNA PCR (-) IgG Ab against VZV	acyclovir ASA	N/A
18	Elbishari et al.[26]	1, M	N/A	5 months	N/A	upper extremity monoparesis intermittent ataxia	globus pallidus	MCA ACA	L	N/A	(-) VZV-DNA PCR	N/A	N/A
19	Bertamino et al.[27]	6, F	R	7 months	unvaccinated	none	thalamus	none	L	(+) IgG Ab against VZV	(+) IgG Ab against VZV (-) VZV-DNA PCR	acyclovir ASA methylprednisolone	N/A
20	O’Reilly et al. [28]	1, M	N/A	5 months	unvaccinated	upper extremity monoparesis ataxia	globus pallidus	MCA ACA	L	N/A	(+) VZV-DNA PCR	acyclovir ASA methylprednisolone	N/A
21	Bertanimo et al. [29]	5, F	N/A	9 months	unvaccinated	none	basal ganglia	none	N/A	N/A	(+) IgG Ab against VZV (-) VZV-DNA PCR low protein content	acyclovir ASA glucocorticoids	N/A
22	Bertanimo et al. [29]	4, M	N/A	8 months	unvaccinated	none	frontal lobe basal ganglia	ICA MCA (M1) ACA (A1)	R	N/A	(+) VZV-DNA PCR	acyclovir ASA prednisone	N/A
23	Schwidetzky et al. [30]	6,F	L	4 months	N/A	hemiparesis	N/A	MCA (M1)	R	N/A	(+) VZV-DNA PCR (+) IgG Ab against VZV	acyclovir methylprednisolone	N/A
24	Our patient	8, M	R	6 months	Unvaccinated	headaches transient facial weakness slurred speech tongue deviation	putamen internal capsule subcortical white matter	none	L	(+) IgG Ab against VZV marginally negative IgM Ab against VZV	(+) VZV-DNA PCR	acyclovir ASA glucocorticoids	2 weeks

## Discussion

### The relationship between VZV and cerebrovascular events

Central nervous system (CNS) involvement is a possible consequence of reactivation of latent VZV infection, as in HZ, as well as, less commonly, a delayed complication of primary VZV infection.

**Fig. 4:**
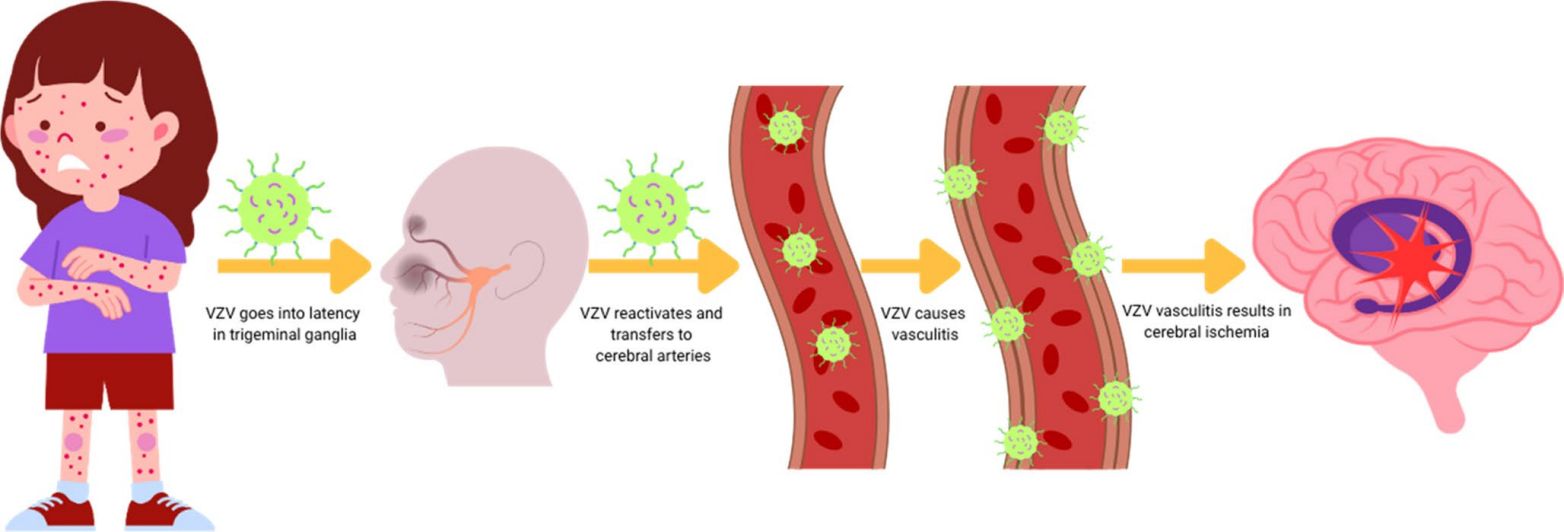
Pathophysiology of post-varicella neurological deficits amongst pediatric patients. After primary infection, the virus goes into latency inside somatosensory ganglia. VZV can reactivate over time under specific circumstances and transfer transaxonally to the cerebral arteries, where it causes transmural inflammation resulting in vascular wall thickening, leading to stenosis and cerebral ischemia. Basal ganglia, a brain region frequently associated with movement disorders, is often affected

VZV has long been associated with cerebral angiopathy and stroke [31]. Its neurotropism, ability to go into latency inside somatosensory ganglia, as well as the possibility of reactivation in immunocompromised individuals is well described [32]. VZV spreads transaxonally to innervated arteries, where it disseminates transmurally, infecting all layers of cerebral vessel wall [31, 33]. This process results in granulomatous arteritis, most frequently involving large arteries of anterior circulation, but small vasculature can be affected as well. The virus causes disruption of internal elastic lamina, progressive thickening of intima and decrease in smooth muscle cells within the media (Fig. 4). VZV’s role in cerebral vasculitis has been confirmed by histopathological evidence of VZV deoxyribonucleic acid (DNA) presence inside biopsied arterial walls. Additionally, VZV can cause protein S deficiency resulting in transient thrombophilia which also contributes to increased risk of cerebral ischemia and can cause acute demyelination in CNS structures [31, 34].

Epidemiological data further supports the role of VZV infection in cerebrovascular events. In adults, ophthalmic-distribution zoster, as well as HZ in other locations was notably associated with higher stroke risk when compared to healthy controls [35, 36]. Numerous case studies and series linking chickenpox infection to arterial ischemic stroke (AIS), especially in children, have been published [37–40]. In the Vascular effects of Infection in Pediatric Stroke II study, nearly 10% of pediatric AIS patients had serological evidence of recent VZV infection, with 16% of those cases attributed to vasculitis [41]. 

The temporal relation between primary infection and neurovascular manifestations of VZV infection remains varied, ranging from less than 1 month to even 9 months [29, 31, 39, 40, 42, 43]. Analysis of searched VZV-related chorea cases showed that the mean time from varicella to neurological manifestations was 5.18 months. Consistent with this epidemiological data, our patient experienced neurological manifestations 6 months after primary infection, with positive serum IgG and borderline negative IgM titers further supporting the role of VZV infection. The lack of history of rash in some patients further complicates establishing VZV as a causative factor of vasculitis, potentially rendering the condition underdiagnosed [43, 44].

### Chorea in pediatric population

Although there are no large-scale studies assessing the incidence of chorea in the general population, it is considered a relatively rare neurological symptom [45]. Etiology of chorea varies between adult and pediatric population, with the main causes of chorea in the adult population including Huntington’s disease (HD) and HD-like syndromes, dopaminergic drug use, cerebrovascular events such as hemorrhagic or ischemic stroke, Wilson disease and systemic autoimmune or infectious disorders. In the pediatric population, Sydenham’s chorea, extrapyramidal variant of cerebral palsy, encephalitides, as well as variety of genetic conditions, including benign hereditary chorea and inherited metabolic disorders, are considered the most prominent etiologies of chorea [13, 46–48]. Given the presence of additional focal symptoms, uncharacteristic of Sydenham’s chorea (central facial nerve palsy, tongue deviation), the lack of history of Group A Streptococcus (GAS) infection, and positive VZV-DNA in CSF assay, both GAS serodiagnostic pathway and throat swab were omitted in our patient.

Vascular hyperkinetic movement disorders are scarcely reported in the pediatric population [46, 49–52]. VZV-vasculitis can lead to seizures, cognitive impairment, as well as various focal deficits [11], but it remains an exceptionally rare etiology of chorea, with only a few cases published in the literature. The proposed pathomechanism involves ischemic damage to subcortical areas, including basal ganglia [53]. 

### Radiological and laboratory findings in VZV cerebral vasculitis

Radiological findings in VZV vasculitis include ischemic lesions located most frequently in the basal ganglia, internal capsule, thalamus, cerebral cortex, subcortical white matter or, characteristically, at the gray-white matter junction. Hemorrhagic foci may also be present; however, they are less frequently reported [43, 54, 55]. Lesions can be multifocal, and may present with post-contrast signal enhancement. Among 20 VZV-related chorea cases containing MRI descriptions, the most frequently affected structure were the basal ganglia (80%), which is consistent with proposed pathomechanism of chorea, as well as with radiological findings in our case [53]. Vascular imaging, in the form of computed tomography-angiography (CTA), magnetic resonance-angiography (MRA) or digital subtraction angiography (DSA), can demonstrate stenosis of large cerebral arteries with “beading” appearance. Vasculopathic changes are present predominantly in anterior circulation, but they may affect the vertebrobasilar system or posterior circulation as well [56–58]. In a series of 21 VZV-related chorea cases with MRA performed, vascular pathology most frequently affected the middle cerebral artery (MCA) (77%) and anterior cerebral artery (ACA) (54%), whereas the posterior cerebral artery (PCA) was affected in 1 case. Nonetheless, 14% of patients lacked significant vascular abnormalities, which was also the case in our patient’s vascular imaging. This might be explained by the presence of exclusively small-vessel vasculitis, with the size of affected vessels being smaller than the resolution of imaging techniques [8, 29, 31, 59, 60]. 

As aforementioned radiological findings can be nonspecific for VZV vasculopathy, it is crucial to correlate imaging studies with laboratory evidence. In a study by M.A. Nagel et al., all 30 patients with VZV-vasculitis were positive for either CSF VZV-DNA or CSF anti-VZV IgG. Within our analysed cases, CSF VZV-DNA PCR was positive in 6 out of 12 instances. Additionally, intrathecal anti-VZV antibody synthesis was present in 5 out of 6 cases that reported this finding. All of the patients in whom both tests were reported were positive for at least one of them, further rendering these CSF findings, alongside the evidence of ischemic lesions in neuroimaging as reliable criteria supporting the diagnosis of VZV-vasculitis [43]. Serum anti-VZV IgG antibodies were detected in all of 7 cases that included the test, although the specificity of this test remains questionable with increasing implementation of routine varicella vaccination [61]. 

### Therapeutic approach to VZV vasculitis and stroke

Literature highlights that management of Varicella‑Zoster Virus (VZV) vasculitis in the context of neuroinfection should be centered on antiviral and immunosuppressive therapy. Intravenous acyclovir at 10–15 mg/kg 3 times a day for up to 2 weeks, often combined with a short course of oral prednisone or intravenous methylprednisolone to target vascular inflammation, has proven to be effective in numerous published cases [8, 12, 27, 31, 62]. However, in a cohort of 19 patients with VZV-vasculitis related chorea, for whom mode of treatment was available, only 11 (58%) were started on acyclovir, while 12 (63%) received glucocorticoids. Combined therapy with both acyclovir and glucocorticoids was administered in 10 cases (53%), including our patient.

The diagnosis of AIS in our patient was followed by the initiation of ASA therapy in the prophylactic dose of 75 mg which is in line with current guidelines and recommendations [54, 55]. In a smaller subset of only 7 (37%) other VZV chorea cases, the use of ASA in combination with antiviral and anti-inflammatory therapy was reported. Since patients with VZV-vasculitis are at an increased risk of stroke recurrence, antiplatelet medication in our patient was continued until clinical improvement and lesion regression was observed, indicating that antiviral and steroid therapy successfully targeted the underlying inflammatory and thrombotic process [18]. 

Symptomatic treatment for chorea was reported in 5 cases (28%) with haloperidol and valproic acid used in 2 of each cases, and carbamazepine in 1 case. Given rapid clinical improvement, symptomatic therapy was not initiated in our case.

In a total of 16 identified cases, complete resolution of hyperkinetic symptoms was observed. Although rarely recorded, the average time from symptom onset to complete clinical recovery was 3.25 weeks, which is longer than the recovery time of our patient.

## Conclusions

Vascular causes warrant consideration as potential etiology of acute extrapyramidal symptoms in the pediatric population. VZV vasculitis is a rare complication of primary infection and should be included in differential diagnosis of chorea, especially in children with recent history of chickenpox. A lack of MRA findings suggestive of vasculitis does not exclude the diagnosis. Vaccination against varicella remains a staple in prevention of cerebrovascular sequelae of the disease. Prompt initiation of antiviral, corticosteroid and antithrombotic therapy might have a positive influence on recovery potential of affected patients; however, further large-scale studies are required to determine optimal therapeutic approach.

## Data Availability

The clinical data comes from the collection of the Department of Developmental Neurology, University Clinical Center in Gdańsk, and has been anonymized in the above work.

## Abbreviations

VZV: Varicella Zoster Virus
HZ: Herpes Zoster
CT: Computer Tomography
CSF: Cerebrospinal Fluid
PCR: Polymerase Chain Reaction
MRI: Magnetic Resonance Imaging
IgG: Immunoglobulin G
IgM: Immunoglobulin M
ASA: Acetylsalicylic Acid
CNS: Central Nervous System
DNA: Deoxyribonucleic Acid
AIS: Arterial Ischemic Stroke
HD: Huntington’s Disease
GAS: Group A Streptococcus
CTA: Computed Tomography-Angiography
MRA: Magnetic Resonance-Angiography
DSA: Digital Subtraction Angiography
MCA: Middle Cerebral Artery
ACA: Anterior Cerebral Artery
PCA: Posterior Cerebral Artery
